# Anatomy of the ventricular septal defect in congenital heart defects: a random association?

**DOI:** 10.1186/s13023-018-0861-z

**Published:** 2018-07-18

**Authors:** Meriem Mostefa-Kara, Lucile Houyel, Damien Bonnet

**Affiliations:** 1 Université Paris Descartes, Sorbonne Paris Cité, 149 rue de Sevres, 75004 Paris, France; 2 Congenital and Paediatric Cardiology, Centre de Référence Malformations Cardiaques Congénitales Complexes - M3C, Necker Hospital for Sick Children, Assistance Publique des Hôpitaux de Paris, Paris, France; 3 Congenital Cardiac Surgery, Hôpital Marie-Lannelongue, Centre de Référence Malformations Cardiaques Congénitales Complexes - M3C, Université Paris-Sud, 133 avenue de la Résistance, 92350 Le Plessis-Robinson, France

**Keywords:** Ventricular septal defect, Congenital heart defect

## Abstract

**Background:**

A ventricular septal defect (VSD) is an integral part of most congenital heart defects (CHD). To determine the prevalence of VSD in various types of CHD and the distribution of their anatomic types.

**Methods:**

We reviewed 1178 heart specimens with CHD from the anatomic collection of the French Reference Centre for Complex Congenital Heart Defects. During the morphologic study a special attention was paid to the localisation of the VSD viewed from the right ventricular side. The VSDs were classified as muscular, central perimembranous, outlet located between the two limbs of the septal band, and inlet. The specimens were classified according to the 9 categories and 23 subcategories of the anatomic and clinical classification of CHD1 (ACC-CHD).

**Results:**

Ventricular septum was almost always intact in anomalies of pulmonary veins (4/73, 5%), Ebstein anomaly (3/21, 14%), and double-inlet right ventricle (DIRV, 1/10, 10%). There was always a VSD in tetralogy of Fallot and variants (TOF, 123 cases) and common arterial trunk (CAT, 55 cases), always of the outlet type. There was almost always a VSD in double inlet left ventricle (33/34, 97%, always muscular), congenitally corrected transposition of great arteries (ccTGA, 23/24, 96%), interrupted aortic arch (IAA, 25/27, 93%), and double outlet right ventricle (DORV, 92/106, 87%). A VSD was found in 68% of aortic coarctation (CoA, 43/63), 62% of heterotaxy syndromes (21/34), 54% of transposition of the great arteries (TGA, 104/194). The VSD was located between the two limbs of the septal band in 100% of TOF and CAT, 80% of IAA, 77% of DORV, 82% of DD. The VSD was of the inlet type in 17% of cc TGA and in 71% of heterotaxy syndromes. In TGA, the VSD was outlet in 40%, central perimembranous in 25%, muscular in 25%, inlet in 10%. In CoA, the VSD was outlet in 44%, central perimembranous in 35%, muscular in 21%. In the 10% hearts with isolated VSD, the distribution was outlet in 44%, central perimembranous in 36%, muscular in 18%, and inlet in 2%.

**Conclusion:**

The anatomic distribution of VSD is similar in isolated VSD, CoA and TGA, while the VSD is predominantly outlet in outflow tract defects except TGA. This reinforces the allegedly different mechanisms in TGA and cardiac neural crest defects. This anatomic approach could provide new insights in the grouping and aetiology of CHD.

## Background

Separation of the left and right ventricles in the human heart is dependent on a single structure, the interventricular septum. This structure is complex from an anatomic standpoint and has multiple embryologic origins. Abnormal completion of this structure during embryonic and fetal life results in a ventricular septal defect (VSD). Isolated VSDs are the most frequent congenital heart defects and are also an integral part of more complex cardiac anomalies [1–3]. It is thus the most common phenotype in all congenital heart defects.

In a previous anatomic study, we demonstrated that the VSD in cardiac neural crest defects is always an outlet VSD, located between the 2 limbs of the septal band, with anatomic variants according to the degree of rotation of the outflow tract [4]. We decided to extend this anatomic study to all types of CHD, in order to search for a link between the anatomic type of the VSD and the overall cardiac phenotype. The objectives of our study were thus: to establish the prevalence of VSD in the main categories of CHD, determined according to the Anatomic and Clinical Classification of Congenital Heart Disease [5]; within each group of CHD, to determine the prevalence of the four main anatomic types of VSD; to classify the CHD according to the presence and the anatomic type of VSD; and to look, for each anatomic type of VSD, for the major phenotypic associations.

### VSD classification

The ventricular septal defects can have different phenotypes. To be able to make a clear distinction between these phenotypes is essential for a correct diagnosis and medical and surgical management. The problem resides in the different systems of nomenclature that continue to create issues in communication between physicians. In the past, different names have been used for the same phenotypes, making the VSD classification still unclear. The same anatomic defect thus continues to be named in different ways at different centers. The history and the evolution of the various systems of classification of VSDs are well explained in a recent review [6]. Indeed, since the late 1980s until now, two different approaches emerged for classification. One is based on the geography of the defect, looking at the location of the VSD within the ventricular septum [7, 8]. The other one is based on the borders of the defect, namely the existence or not of a fibrous component at the postero-inferior part of the defect. This is important for the surgeon because of the risk of injury of the conduction tissue if such a fibrous component exists [9]. The differences between these two approaches still induce confusion in the classification and nomenclature of the VSDs.

In our anatomic study, we have decided to use primarily the geographic approach, that is the anatomic location of the VSD viewed from the right ventricle, while taking into account the borders to define the subtypes, keeping in mind these ongoing differences and classification issues. The geographic approach seemed to us more suited to our daily clinical practice in our surgical centers [10].

## Methods

### Material

We have examined 1178 human heart specimens with CHD from the anatomic collection of the French Reference Center for Complex Congenital Heart Defects (M3C). Hearts without structural heart anomaly were excluded from the study. We classified these specimens in ten groups according to the anatomic and clinical classification of congenital heart diseases ACC-CHD [5]. This classification is exhaustive and in order to be more pertinent, we chose the subgroups that are the most frequently encountered in clinical practice of pediatric and congenital cardiology as shown in the Table 1.

**Table 1:** Distribution of the VSDs according to the ACC-CHD classification

ACC-CHD classification	Nb specimens	Nb VSD	% nb VSD
Heterotaxy	34	21	61,7
Anomalies of the venous return	34	4	0,5
Anomalies of the atria and interatrial communications	9	0	0
Anomalies of the atrioventricular junctions and valves	172	111	64
Complex anomalies of the atrioventricular connections	24	23	96
Functionally univentricular hearts	121	37	30,5
Ventricular septal defects	72	72	100
Anomalies of the ventricular outflow tracts	558	387	69
Anomalies of the extrapericardial arterial trunks	107	72	67
Congenital anomalies of the coronary arteries	8	2	25
Total	1178	729	62

We studied 34 hearts with heterotaxy syndrome, 73 anomalies of the venous return, 9 anomalies of the atria and interatrial communications, 172 anomalies of the atrioventricular junctions and valves, 24 complex anomalies of the atrioventricular connections, 121 functionally univentricular hearts, 72 isolated ventricular septal defects, 558 anomalies of the ventricular outflow tracts, 107 anomalies of the extra pericardial arterial trunks and 8 anomalies of the coronary arteries.

### Methods

The intracardiac anatomy of each heart specimen was carefully studied. We separated the hearts into two groups depending on the presence or not of a VSD. When a VSD was present, we paid particular attention to its anatomic characteristics, analyzed from the right ventricular side. We specified the phenotype of the VSD with its exact localization within the ventricular septum, according to the geographic approach. Then, the borders of the VSD were carefully described, as well as the relationship between the atrioventricular valves and the arterial valves.

**Fig. 1:**
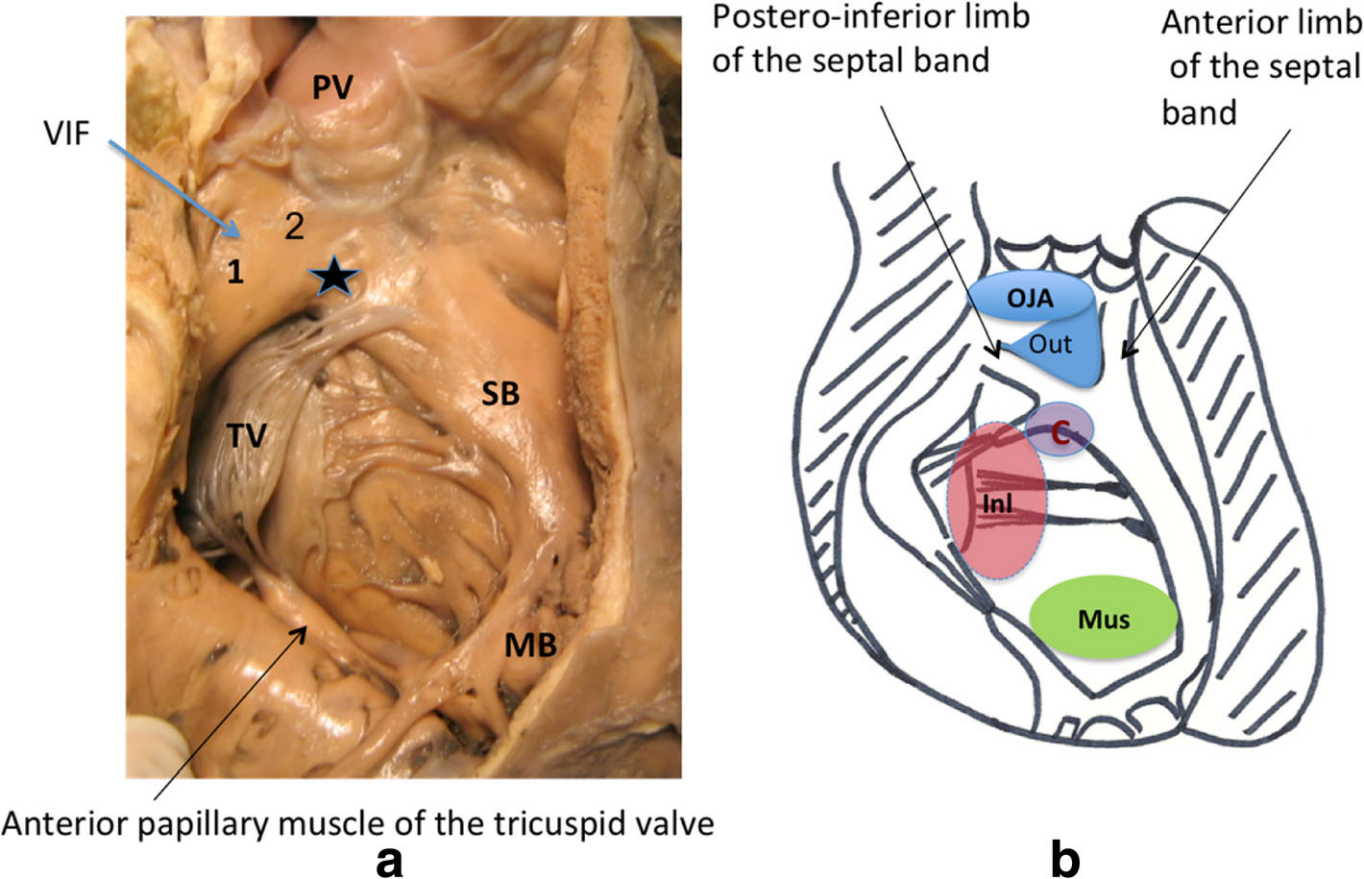
**a**: Right ventricular aspect of the ventricular septum in a normal heart. The muscular bands of the right ventricle are: the ventriculo-infudibular fold (VIF, including parietal band [5] and subpulmonary conus [1]); the outlet septum (star), the septal band or septomarginal trabeculation (SB) with its two limbs, anterosuperior and postero-inferior, the moderator band (MB), the anterior papillary muscle of the tricuspid valve. PV: pulmonary valve; TV: tricuspid valve. **b**: represent the four main anatomic types of ventricular septal defects viewed from the right ventricle. C: central VSD, OJA: outlet juxta arterial VSD, Inl: Inlet VSD, M: muscular VSD, Out: outlet VSD, T: tricuspid Valve, VIF: ventriculo-infundibular fold

In order to fully describe the morphologic features of the VSD, we should first to clearly define the terminology used to characterize the different parts of the right ventricle (Fig. 1).

#### Normal anatomy of the right ventricle and localization of the VSDs

The ventriculo-infundibular fold (grouping together parietal band and subpulmonary conus) is a muscular band, which separates the anterior leaflet of the tricuspid valve from the leaflets of the pulmonary valve in the normal heart [11–13]. The left extremity of the ventriculo-infundibular fold represents the upper part of the ventricular septum: the outlet (or conal) septum, which is fused with the upper extremity of the septal band (or septomarginal trabeculation), between its two limbs, antero-superior and postero-inferior. In the normal heart, the postero-inferior limb of the septal band, which carries the papillary muscle of the conus and its attachments, is not fused with the ventriculo-infundibular fold, but is separated from it by the atrioventricular part of the membranous septum, connecting the anterior leaflet of the tricuspid valve with the leaflets of the aortic valve [7]. The interventricular part of the membranous septum connects the septal leaflet of the tricuspid valve with the right coronary and the non-coronary leaflets of the aortic valve.

#### Classification of the VSDs

We chose to classify the VSDs into four groups according to their localization within the right ventricular septum [10] (Fig. 1).

1- Outlet VSDs are opening to the outlet of the right ventricle and located between the two limbs of the septal band [4]. There are some variations observed especially regarding the postero-inferior rim of the VSD that can be fibrous (with fibrous continuity between the aortic and the anterior leaflet of the tricuspid valve: malalignment outlet VSD with perimembranous extension) or muscular, due to a fusion between the posterior limb of the septal band with the ventriculo-infundibular fold. The other anatomical variation is the muscular or fibrous nature of the outlet (conal) septum: when the outlet septum is muscular, the VSD is a malalignment type outlet VSD; when the outlet septum is fibrous or absent the VSD is a doubly committed or juxta-arterial outlet VSD [4] .

2-Inlet VSDs are located in the postero-inferior part of the interventricular septum, below the atrioventricular junction. These VSDs can be immediately adjacent to the AV valves with mitral-to-tricuspid fibrous continuity if there are two separate AV junctions, or can have entirely muscular borders: inlet muscular defects.

There are three types of inlet VSDs: inlet VSD in the setting of a common atrioventricular junction (atrioventricular septal defect), inlet VSD associated with straddling and overriding tricuspid valve, with malalignment between the atrial and the ventricular septum, and the inlet muscular VSD.

3- Trabecular muscular VSDs are located within the trabecular septum and have entirely muscular borders.

4- Central perimembranous VSDs are located below the posterior limb of the septal band and under the ventriculo-infundibular fold, at the site of the interventricular part of the membranous septum. This type of VSD always has a fibrous postero-inferior rim, but the fibrous continuity between the tricuspid and the aortic valves concerns the septal leaflet of the tricuspid valve, and not the anterior leaflet as in malalignment VSD with perimembranous extension [4].

When there was more than one VSD, we chose the main type of the VSD as outlet, inlet or central perimembranous.

The first objective of the study was to establish the prevalence of the four main anatomic types of VSD (outlet, inlet, muscular, central perimembranous) in the ten main groups of ACC-CHD, and thus in the most important subgroups. We then classified the outlet and inlet VSDs according to the anatomical details of their borders as described above.

In order to simplify the presentation of the results, we chose to separate the CHD into four groups, according to the frequency of associated VSD and their anatomic type: VSD constant of a single type, VSD non constant of a predominant type, VSD non constant of variable type andVSD rare. A fifth group, including isolated VSD, was considered.

### Statistical analysis

Statview° software was used for data analysis. The qualitative anatomic variables were presented with percentages. A chi-square test analysis was used to evaluate possible differences between the parameters. Statistical significance was assessed by using a cutoff *p* value of 0.05.

## Results

### Prevalence of the VSD in all specimens

A VSD was present in 729 heart specimens with CHD (62% of all hearts). The main VSD was muscular in 98 specimens (14%), outlet in 391 (53%), inlet in 149 (21%) and central perimembranous in 91 (12%). Tables 1 and 2 show the prevalence of the VSD in the groups of ACC-CHD, and Table 3 shows the prevalence of VSDs in the main subgroups of CHDs.

**Table 2:** Distribution of the VSD’s type according to ACC-CHD classification

ACC-CHD classification	Nb VSD	Central perimembranous	Outlet	Inlet	Muscular
Heterotaxy	21	1	1	19	0
Anomalies of the venous return	4	1	1	0	2
Anomalies of the atria and interatrial communications	0	0	0	0	0
Anomalies of the atrioventricular junctions and valves	111	4	0	102	5
Complex anomalies of the atrioventricular connections	23	4	15	0	4
Functionnally univentricular hearts	37	1	0	1	35
Ventricular septal defects	72	26	32	0	14
Anomalies of the ventricular outflow tracts	387	34	301	15	37
Anomalies of the extrapericardial arterial trunks	72	19	41	0	12
Congenital anomalies of the coronary arteries	2	1	0	0	1

**Table 3:** VSD localization in the main subgroups of CHDs

CHD	VSD localization				
	Nb of VSD	Outlet	Inlet	Central perimembranous	Muscular
Tetralogy of Fallot and variants	123	123	0	0	0
Common arterial trunk	55	55	0	0	0
Double inlet left ventricle	33	0	0	0	33
Common atrioventricular canal	85	0	85	0	0
Double outlet right ventricle	92	71	13	4	4
Interruption of the aortic arch	25	20	0	2	3
Congenitally corrected T GA	23	15	4	4	0
Heterotaxy syndrome	21	1	19	1	0
Coarctation	43	19	0	15	9
Transposition of great arteries	104	42	9	27	26
Double inlet right ventricle	1	0	1	0	0
Coronary anomalies	2	0	0	1	1
Ebstein anomaly	3	0	0	1	2
Isolated VSD	72	32	1	26	13

### Distribution of the VSD phenotype according to the 5 groups mentioned above

#### VSD constant and of a single type:

A VSD was always present in hearts with tetralogy of Fallot and variants (TOF, 123), common arterial trunk (CAT, 55), double inlet left ventricle (DILV, 33) and in complete atrioventricular septal defect (complete AVSD, 85). Variants of TOF included TOF with pulmonary stenosis, with pulmonary atresia, and with absent pulmonary valve.

In TOF and variants and CAT, the VSD was always an outlet VSD, located between the two limbs of the septal band. When we looked at the rims of the VSD, there was a fibrous continuity between the aortic valve and the anterior leaflet of the tricuspid valve in 69/123 hearts (56%) in TOF and variants, and in 1/55 hearts (1,8%) in CAT. In hearts with double inlet left ventricle the VSD was also constant and always muscular. In hearts with complete AVSD, the VSD was always an inlet VSD in the setting of a common atrioventricular junction.

#### VSD non-constant, with a predominant type:

The VSD was almost always present in hearts with congenitally corrected TGA (cc-TGA, 23/24; 96%), interruption of the aortic arch (IAA, 25/27; 93%), double outlet right ventricle (DORV, 92/106; 87%), and in heterotaxy syndromes (HTX, 21/34; 62%).

In hearts with cc-TGA, the VSD was most often an outlet VSD (19/23; 82%) and central perimembranous in 4(17%). In hearts with heterotaxy syndrome, the VSD was predominantly an inlet VSD (15/21; 71%). In hearts with IAA, the VSD was predominantly an outlet VSD (20/25; 80%), and in DORV as well (71/92; 77%).

#### VSD non-constant, of variable type:

This group includes hearts with transposition of the great arteries (TGA) and coarctation.

In hearts with TGA, the VSD was present in 54% (104/194) of the specimens and the anatomical type of the VSD was variable: outlet in 42/104(40%), central perimembranous in 27/104(26%), muscular in 26/104(25%) and inlet in 9/104 (9%).

In hearts with coarctation, the VSD was present in 68% (43/63) of heart specimens and the anatomical type of the VSD was also variable: outlet in 19/43 (44%), perimembranous or central in 15/43 (35%), muscular in 9/43 (25%), and none of the inlet type.

#### VSD rare

The VSDs were rarely encountered in the anomalies of the pulmonary veins (4/73, 5%), Ebstein anomaly (3/21, 14%), double inlet right ventricle (1/10, 10%) and in coronary anomalies (2/8, 25%).

### Distribution of the VSD phenotypes in isolated VSD

The VSD was isolated in 10% of all hearts with VSD (72/729). In this group**,** 44% have an outlet VSD, 36% a central perimembranous VSD, 18% a muscular VSD and 2% an inlet VSD.

### Description of the VSDs according to their borders (Table 4)

Among the 391 hearts with an outlet VSD (53% of all the VSDs), there was a fibrous continuity between the anterior leaflet of the tricuspid valve and the aortic valve in 108 hearts (30%). The proportion of outlet VSD with fibrous continuity was different according to the main CHD: 68/123 (55%) in TOF and variants, 4/71 (4%) in double outlet right ventricle, none in interruption of aortic arch, 23/32 (71%) in isolated outlet VSD. In the common arterial trunk, the borders of the VSD were always muscular except in one heart.

**Table 4:** Description of the VSDs according to their localization within the ventricular septum and to their borders

Types of VSDs according to their localization	VSDs (Nb)	Nb of VSDs with Fibrous continuity	Nb of VSDs with muscular borders	Fibrous continuity (%)
Central perimembranous	91	91	0	100%
Outlet	391	108	283	28%
Inlet	149	137	12	91%
Common AV junction type	119	119	0	100%
Malalignement of the atrial and ventricular septum	18	18	0	100%
Inlet muscular	12	0	12	0%
Muscular	98	0	98	0%

Among the 149 hearts with an inlet VSD, there was a fibrous continuity between the tricuspid and the mitral valve in 137 (90%).

### Type of CHD according to the anatomic type of VSD

Outlet VSDs were found in 70% of the cases in association with outflow tract defects: neural crest defects in 60% (TOF and variants, CAT, IAA and DORV), and TGA in 11%. Outlet VSDs were isolated in 8% and associated in 4% with ccTGA.

Inlet VSDs are mainly found in complete AVSD (85/149, 57%), in heterotaxy syndromes with common AV canal (19/149, 13%). Straddling tricuspid valve accounts for 6% (9/149) of the inlet VSDs.

Trabecular muscular VSDs are mainly encountered in double inlet left ventricle (33/98, 34%) and TGA (26/98, 26%). They were isolated in 14% of the cases.

Central perimembranous VSDs are isolated in 28% of cases, associated with TGA in 27% (27/91) and with aortic coarctation in 16% (15/91).

## Discussion

### Prevalence of the VSD

In this anatomical study in 1178 human heart specimens, the prevalence of VSD, isolated or in the setting of more complex CHD is 62%. This result confirms that the VSD is indeed part of the cardiac phenotype in the majority of CHDs. The outlet VSD is the predominant anatomic type in our collection, with an incidence of 53%, while trabecular muscular and central perimembranous VSDs were found in only 13 and 12% of the collection. This distribution is markedly different from that seen in epidemiological studies, where isolated VSDs are the most frequent of all CHDs, with trabecular muscular and central perimembranous the most frequent anatomic types [1]. This difference may come from the fact that we are looking at autopsy specimens vs. live cohorts, and that isolated VSDs are thus considerably less frequent than in living populations.

### Distribution of VSD phenotypes

In this study we showed that the distribution of the VSD phenotypes is markedly different depending on the associated CHD. This can either be a logical association, determined by embryology, or a random association with no clear embryological link.

#### Constant VSD:

The VSD is an integral part of the phenotype in some CHDs, which suggests that this association is not a random one. The neural crest defects, including tetralogy of Fallot and variants and CAT, share the same VSD located in the outlet between the two limbs of the septal band, as previously described [4]. This type of VSD is due to the absence of normal fusion between the developing outlet septum and the upper extremity of the septal band during embryonic development, which means that the wedging of the aorta between the two atrioventricular valves is not fully completed. In double inlet left ventricle, the VSD or bulboventricular foramen is always a trabecular muscular VSD [14–17]. This is because this VSD represents the persistent primitive communication between the two developing ventricles, bordered in its upper part by the inner curvature and in its lower part by the crest of the muscular trabeculated septum.

In the same way, it is not surprising that the VSD found in common AV canal defects, is an inlet VSD resulting from the complex embryologic lack of atrioventricular septation caused by the commonality of the atrioventricular junction [18].

These three types of CHD (cardiac neural crest defects, double inlet left ventricle, common atrioventricular canal defects) can be considered the result of an arrest in the normal development of the heart, at various stages: outflow tract rotation and wedging, early looping stage, atrioventricular septation. At these different stages the ventricular septation is still incomplete, and thus the cardiac phenotype obligatorily includes a VSD, whose location depends on the stage at which the development has stopped.

#### Not constant, with a predominant type

This category includes congenitally corrected TGA (VSD 96%, outlet type in 82% of cases), interruption of the aortic arch (VSD 93%, outlet type in 80%), double outlet right ventricle (VSD 87%, outlet type in 77%), and heterotaxy syndromes (62%, inlet type in 71%). Congenitally corrected TGA is a complex laterality heart defect in which the first morphogenetic abnormality occurs at the ventricular level, resulting in discordant atrioventricular relations, associated with TGA. The VSD in the majority of cc-TGA is an outlet VSD, most often with inlet extension, resulting in a fibrous continuity between pulmonary, mitral and tricuspid valves. This extension in the postero-inferior part of the ventricular septum probably results in the malalignment between the atrial and ventricular septum, constant in cc-TGA, except when there is pulmonary atresia, and accounts for the frequent association with straddling tricuspid valve [19]. In clinical series the incidence of associated VSD is less, reflecting the fact that the severe forms of the disease are overrepresented in anatomic collections.

Interruption of the aortic arch without VSD is a rare disease. Moreover, type B and C are considered cardiac neural crest defects, in which the VSD is thus obligatorily an outlet VSD.

Double outlet right ventricle without a VSD is also rare, except when DORV is associated with mitral atresia. The majority of the VSD in DORV are committed to the great vessels, (subaortic, subpulmonary or doubly committed), and of necessity opening in the outlet of the right ventricle. Non-committed VSD can be muscular, central perimembranous or opening in the inlet of the right ventricle, and are much less frequent.

Inlet VSDs are predominant in the setting of heterotaxy syndromes. This reflects the high incidence of common AV junction in heterotaxy.

#### Variable type

Half of the hearts with transposition of the great arteries have an intact ventricular septum. In addition, the VSD in TGA can be of any type: outlet in 40% of cases, inlet, trabecular muscular, or central perimembranous [20]. The fact that the anatomic distribution of VSD is comparable to that found in isolated VSDs and in coarctation indicates a likely random association. This reinforces the hypothesis of different genetic mechanisms involved in the two main categories of outflow tract defects: TGA and cardiac neural crest defects. In the latter, the VSD is constant and always of the outlet type, in the former it is non constant and of variable type. Indeed, genetic studies in human families, as well as experimental studies in animal models, indicate that “laterality genes” like Nodal and ZIC3 are involved in pathogenesis of cc-TGA and heterotaxy syndromes, but also in TGA [21]. By contrast, cardiac neural crest defects are frequently associated with 22q11 microdeletion, which is exceptional in patients with TGA.

One can hypothesize that the embryologic mechanism of the “main” defect in TGA or in coarctation does not cause simultaneously the VSD and the main defect in all cases. Undoubtedly, there might be non-random association in coarctation with outlet VSD and posterior displacement of the conal septum. But our data suggest that classifying heart defects according to their supposed embryological mechanism is not possible in all anatomical groups.

### Limitations of the study

As underlined before, the distribution of the CHD is very different in an anatomic collection of heart specimens, compared to in-hospital and epidemiologic studies. The complexity of the defects is of necessity higher in heart specimens.

Categorization of the anatomical type of the VSD using the geographical classification was easy for the majority of the specimens, but for some hearts the anatomical type of the VSD was difficult to determine. However, the subtleties of anatomic landmarks are even more difficult to recognize in living patients, particularly on echocardiography. Some VSDs are a combination of two anatomic subtypes, for example in some complex DORV with a huge VSD extending from the inlet to the outlet.

## Conclusion

The VSD is an integral part of the cardiac phenotype in some CHDs (cardiac neural crest defects, common AV canal, heterotaxy syndromes), indicating a logical and not random association, related to an arrest in normal heart development.

In TGA and CoA, the VSD is not constant and the anatomic distribution of VSD is similar to that in isolated VSDs, indicating a likely random association or different mechanisms for the different associations.

This original approach, based on the anatomic characteristics of one part of the cardiac phenotype, namely the VSD in our study, could provide new insights in grouping and etiology of CHDs. It could be extended to other parts of the phenotype, in order to improve the diagnostic process, and to better understand the links between the cardiac phenotype and the morphogenesis of CHDs.

## Abbreviations

AVSD: atrioventricular septal defect
CAT: common arterial trunk
CC TGA: congenitally corrected transposition of great arteries
CHD: congenital heart defect
Coa: coarctation
DILV: double inlet left ventricle
DORV: double outlet right ventricle
HTX: hétérotaxie
IAA: interruption aortic arch
TGA: transposition of great arteries
TOF: tetralogy of Fallot
VSD: ventricular septal defect

## References

[1] Khoshnood B, Lelong N, Houyel L (2012). Prevalence, timing of diagnosis and mortality of newborns with congenital heart defects: a population-based study. Heart.

[2] Dolk H, Loane M, Garne E (2011). European surveillance of congenital anomalies (EUROCAT) working group. Congenital heart defects in Europe: prevalence and perinatal mortality, 2000 to 2005. Circulation.

[3] Mitchell SC, Korones SB, Berendes HW (1971). Congenital heart disease in 56,109 births. Incidence and natural history. Circulation.

[4] Mostefa-Kara M, Bonnet D, Belli E, Fadel E, Houyel L. Anatomy of the ventricular septal defect in outflow tract defects: similarities and differences. J Thorac Cardiovasc Surg. 2015;149(3) 10.1016/j.jtcvs.2014.11.087. 10.1016/j.jtcvs.2014.11.087 25703407

[5] Houyel L, Khoshnood B, Anderson RH (2011). Population-based evaluation of a suggested anatomic and clinical classification of congenital heart defects based on the international Paediatric and congenital cardiac code. Orphanet J Rare Dis.

[6] Bailliard F, Spicer DE, Mohun TJ, Henry GW, Anderson RH (2015). The problems that exist when considering the anatomic variability between the channels that permit interventricular shunting. Cardiol Young.

[7] Soto B, Ceballos R, Kirklin JW (1989). Ventricular septal defects: a surgical viewpoint. J Am Coll Cardiol.

[8] Van Praagh R, Geva T, Kreutzer J (1989). Ventricular septal defects: how shall we describe, name and classify them?. J Am Coll Cardiol.

[9] Spicer DE, Hsu HH, Co-Vu J, Anderson RH, Fricker FJ (2014). Ventricular septal defect. Orphanet J Rare Dis.

[10] Houyel L, Rickert-Sperling S, Kelly R, Driscoll D (2016). Molecular pathways and animal models of ventricular septal defect. Congenital heart diseases: the broken heart.

[11] Van Praagh R (2009). The first Stella van Praagh memorial lecture: the history and anatomy of tetralogy of Fallot. Semin Thorac Cardiovasc Surg Pediatr Card Surg Annu.

[12] Anderson RH, Weinberg PM (2005). The clinical anatomy of tetralogy of fallot. Cardiol Young.

[13] Anderson RH, Jacobs ML (2008). The anatomy of tetralogy of Fallot with pulmonary stenosis. Cardiol Young.

[14] Cook AC, Anderson RH (2006). The anatomy of hearts with double inlet ventricle. Cardiol Young.

[15] Wilkinson JL, Anderson RH (2012). Anatomy of functionally single ventricle. World J Pediatr Congenit Heart Surg.

[16] Vanpraagh R, Ongley PA, Swan HJ (1964). Anatomic types of single or common ventricle in man. Morphologic and geometric aspects of 60 necropsied cases. Am J Cardiol.

[17] van Praagh R, Plett JA, van Praagh S (1979). Single ventricle. Pathology, embryology, terminology and classification. Herz.

[18] Kim JS, Virágh S, Moorman AF, Anderson RH, Lamers WH (2001). Development of the myocardium of the atrioventricular canal and the vestibular spine in the human heart. Circ Res.

[19] Hosseinpour A-R, McCarthy KP, Griselli M, Sethia B, Ho SY (2004). Congenitally corrected transposition: size of the pulmonary trunk and septal malalignment. Ann Thorac Surg.

[20] Boesen I (1963). Complete transposition of the great vessels: importance of septal defects and patent ductus arteriosus. Analysis of 132 patients dying before age 4. Circulation.

[21] Unolt M, Putotto C, Silvestri LM (2013). Transposition of great arteries: new insights into the pathogenesis. Front Pediatr.

